# The cyclic interaction between daytime behavior and the sleep behavior of laboratory dogs

**DOI:** 10.1038/s41598-021-04502-2

**Published:** 2022-01-10

**Authors:** Ivana Gabriela Schork, Isabele Aparecida Manzo, Marcos Roberto Beiral De Oliveira, Fernanda Vieira da Costa, Robert John Young, Cristiano Schetini de Azevedo

**Affiliations:** 1 School of Sciences, Engineering and Environment, University of Salford Manchester, Salford, UK; 2 Programa de Pós-Graduação em Ecologia de Biomas Tropicais, Departamento de Evolução, Biodiversidade e Meio Ambiente, Universidade Federal de Ouro Preto, Ouro Preto, Minas Gerais Brasil

**Keywords:** Animal behaviour, Behavioural ecology

## Abstract

Sleep deprivation has been found to negatively affect an individual´s physical and psychological health. Sleep loss affects activity patterns, increases anxiety-like behaviors, decreases cognitive performance and is associated with depressive states. The activity/rest cycle of dogs has been investigated before, but little is known about the effects of sleep loss on the behavior of the species. Dogs are polyphasic sleepers, meaning the behavior is most observed at night, but bouts are also present during the day. However, sleep can vary with ecological and biological factors, such as age, sex, fitness, and even human presence. In this study, kennelled laboratory adult dogs’ sleep and diurnal behavior were recorded during 24-h, five-day assessment periods to investigate sleep quality and its effect on daily behavior. In total, 1560 h of data were analyzed, and sleep metrics and diurnal behavior were quantified. The relationship between sleeping patterns and behavior and the effect of age and sex were evaluated using non-parametric statistical tests and GLMM modelling. Dogs in our study slept substantially less than previously reported and presented a modified sleep architecture with fewer awakenings during the night and almost no sleep during the day. Sleep loss increased inactivity, decreased play and alert behaviors, while increased time spent eating during the day. Males appeared to be more affected by sleep fragmentation than females. Different age groups also experienced different effects of sleep loss. Overall, dogs appear to compensate for the lack of sleep during the night by remaining inactive during the day. With further investigations, the relationship between sleep loss and behavior has the potential to be used as a measure of animal welfare.

## Introduction

Sleep disturbances are widely recognized in human medicine as good indicators of both physical and psychological wellbeing^1^, especially considering that the comprehensive study of the sleep/wake cycle, in both human and non-human models, concluded that sleep is a vital part of the homeostatic process^2,3^. Furthermore, sleep in mammals is a species-specific process, meaning its structure is associated with specific environmental cues, such as period of activity, feeding and reproductive cycles^2,4^. Consequently, sleep influences behavior and in return, behavior can influence sleep^5,6^.

Sleep can be identified by three conditions: the presence of a specific posture; reduced but reversible responsiveness; and homeostatic regulation^7^. In addition, sleep has two other important temporal characteristics: its duration in 24 h and the number of bouts of sleep in a 24-h period^7^. Monophasic sleepers are those who sleep in one consistent block, such as humans, whereas polyphasic sleepers are those whose sleep is divided into several blocks during a period, such as giraffes or dogs^8^. However, regardless of the structure, sleep in mammals usually alternates between two phases: non-REM sleep and REM sleep (REM: Rapid-eye movement)^8^. Each phase is responsible for the restoration of different physiological and cognitive functions. Non-REM sleep is connected to the energy homeostasis, the immune system and controls the metabolic functions, while REM-sleep is connected to neural plasticity, memory consolidation and emotional processing^9,10^. Although not all functions of sleep are known, it possesses a high adaptative value, since any changes in its structure will have deleterious consequences on bodily functions^7,10,11^.

Sleep loss can be caused by a total or partial restriction of sleep duration or by sleep fragmentation, which may or may not impact sleep duration, but still has negative consequences^12^. Sleep deprivation has been proven to impair circadian rhythms^13^. In humans, lack of sleep compromises motor and cognitive diurnal functions, increases anxiety and depressive states, and alters consumption behaviors and emotional responses^11,14–16^. Similarly, in others animal models, it affects locomotor patterns and general activity^17–19^, increases the display of aggression^20^ and anxiety-like behaviors^21^, decreases cognitive performance^22^, and has been associated with changes in mating^23^, migration^24^, and communication patterns^25^. Therefore, understanding sleep characteristics and specially changes in sleep are relevant to the health and well-being of animals under human care^26,27^.

Despite several studies analyzing the importance of sleep and the extensive literature on the consequences of sleep loss in both humans and non-human models^28^; presently there is only a basic understanding of the variables that affect sleep in dogs^29^ and specially, how sleep loss affects their behavior.

Domestic dogs have a diurnal circadian rhythm, and their peak of activity occurs during the light period, whereas rest is most frequent at night^30,31^. However, as they are classified as polyphasic sleepers, sleeping bouts are also observed during the day^30,32^. Usually, dogs spend 43–60% of the 24 h sleeping and REM sleep occurs at rates between 20–36% of the total sleeping period^32–34^. Sleeping bouts are observed in rates that vary between 23^35^ and 60^34^ and sleep structure is modified by different biological characteristics such as sex, age, level of activity, feeding regimes, environmental conditions (e.g. light), human activity and even social interactions^26,31,32,36–39^. For instance, studies with humans^40^ and rats^41^ have shown that females have better sleep efficiency and better recovery of episodes of sleep loss than males, and similar results have been found for female dogs^38^.

Likewise, research demonstrated that age affects sleep quantity and quality, with increases in sleep fragmentation, decreases in REM-sleep bouts at night and increases in time in non-REM sleep during the day being associated with increased age^42,43^. The same results are found in dogs^31,34^. Older individuals also exhibit: less REM sleep during the night; longer non-REM bouts during the day; and increased wakefulness during the night. However, the frequency of night sleep bouts tends to decrease with age, resulting in extended sleep duration^29^.

Recently, the association between sleep and activity was investigated in dogs, which found that the macrostructure of individual sleep changes between days where dogs have higher activity threshold and days with lower activity^37^ or passiveness^33^. Pre-sleep activity improved sleeping time, time spent in REM-sleep, reduced latency to first sleep bout and less awakening during subsequent sleep, increasing overall sleep efficiency^33,37^.

Changes in the structure of sleep can also be caused by events experienced during wakefulness^27^. For instance, in humans and rats, sleep disruption is associated with distress^44,45^. Sleep disruption is also considered a stressor by itself, since lack of sleep can overload the adrenal system function, suppressing immunity and leading to depressive states^46,47^; a response observed in rats^48^.

Due to the association between sleep and biological systems and the severe impact of sleep loss on animals´ physical and psychological wellbeing, it is supposed that dogs with disrupted sleeping patterns may suffer from reduced welfare. As investigations on this topic are scarce, research is necessary to elucidate the relationship between sleep disruption and behavior with the species. This study aimed to assess the association between sleep disruption and the patterns of diurnal behavior of laboratory dogs, and what roles factors such as age and sex played in sleep behavior. As the impact of sleep loss is substantial, is unquestionable that an experimental study manipulating the sleep parameters of the dogs would not be ethically justified. Therefore, our study used an observational approach to investigate the natural sleeping patterns of dogs in an environment which is known to promote stress responses – laboratory kennels^49^. This approach has been used before to assess whether sleep can be a measure of welfare in shelter dogs^26^.

## Results

### Overall sleep and activity profiles of dogs

Average nocturnal sleep was 6.1 ± 3.9 h (370 ± 232 min) with a mean of 10.8 ± 7.01 bouts per night. Over 65% of the time, the first sleeping bout occurred between 18:00 and 19:00 h (mean latency to sleep 51 ± 107 min) and the dogs woke up, on average, 20 min (± 24 min) before the morning shift of the kennel started (at 07:00 h). During the day, dogs were recorded sleeping only 6.02% (± 0.28) of the observations with a mean duration of 4 ± 7 min and with less than one bout per day (0.14 ± 0.43).

A Wilcoxon test showed that dogs slept significantly more at night in comparison to the day (Z = 5.65, df = 1, *p* < 0.001), this effect was also observed with the number of sleeping bouts (Z = 31.39, df = 1, *p* < 0.001). Sleep was significantly the most expressed of the three behaviors (sleep, activity, and inactivity) recorded at night (Friedman = 177.5, *p* < 0.001, df = 2).

**Figure 1:**
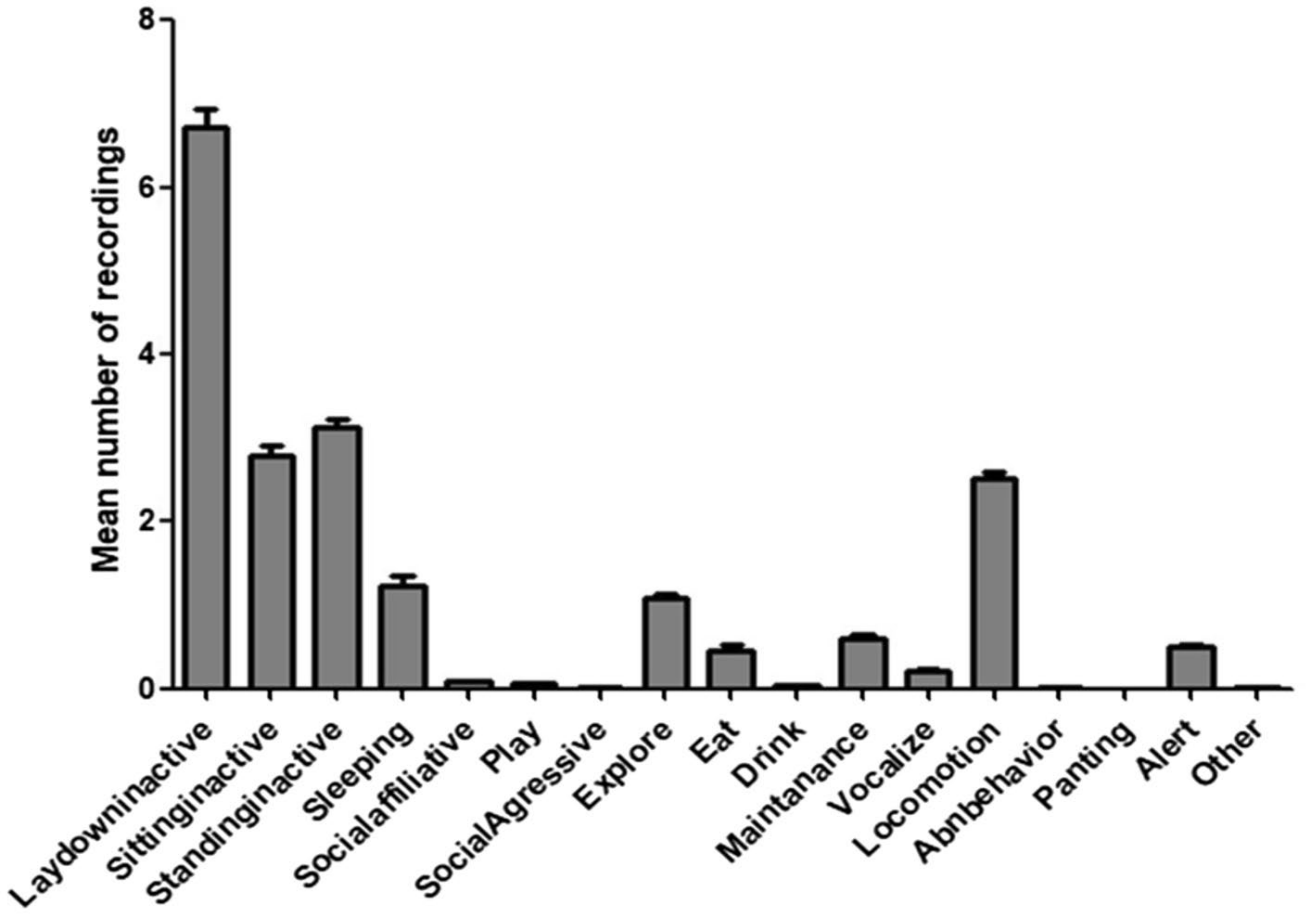
Mean expression of different behavioral categories during diurnal observations of laboratory dogs kenneled at the Federal University of Ouro Preto, Brazil. Error bars show ± standard deviation of the mean.

Although sleep was rarely observed during the day; the amount of time spent inactive during daylight accounted for 61% of total observations [“Sitting inactive” (13.56% ± 4.89), “Standing inactive” (15.12% ± 4.43), “Lay down inactive” (32.64% ± 8.07)]. Inactivity was significantly the most expressed diurnal behavior (Friedman = 1844, *p* < 0.0001, df = 2), followed by locomotion (12.23%), sleeping (6.03%) and exploring (5.22%). All other behaviors were recorded in less than 5% of the observations (Fig. 1).

**Figure 2:**
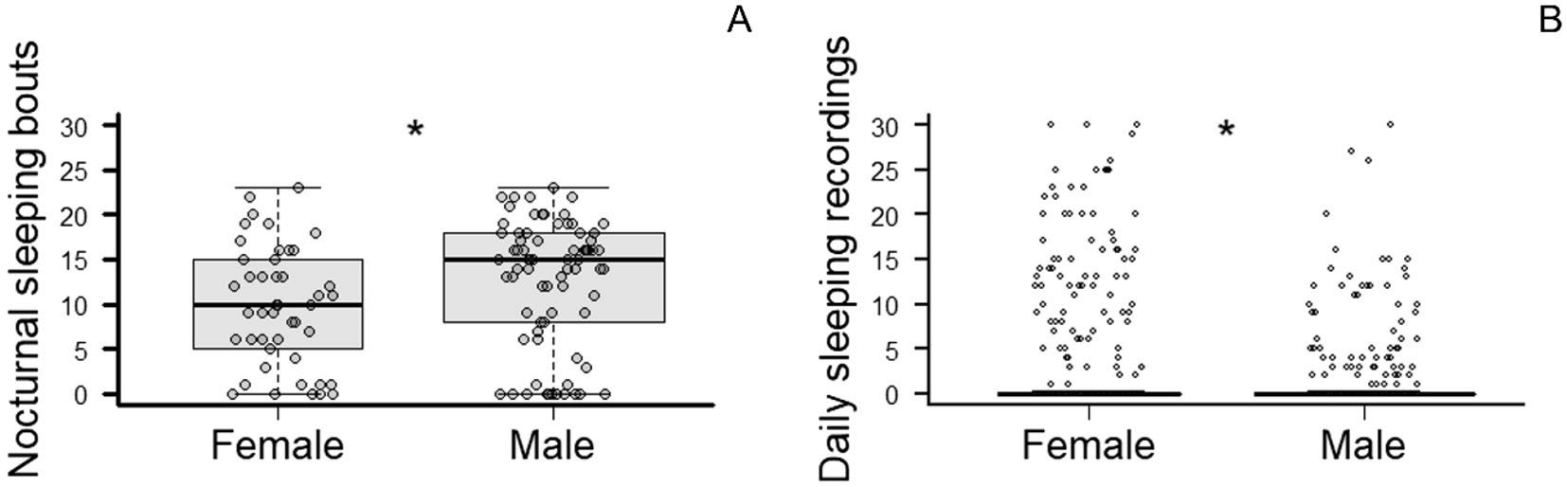
Sex patterns of laboratory dogs’ sleep metrics. (**A**) Number of nocturnal sleeping bouts for female and male dogs. The tick line represents the median, whiskers show maximum and minimum values, the box represents interquartile ranges. *Indicates a significant difference at *p* < 0.05. (**B**) Daily sleep recordings (counts) observed for female and male dogs in randomized 15-min observations with 30-s recording intervals. Tick line represents the median, *Indicates a significant difference at *p* < 0.05. IQR box and whiskers are reduced in the image due to the large number of zeros recorded for daily sleep (See Supplementary Figure S1 for a histogram of the data).

We observed that age and sex affected sleep duration and numbers of bouts, both at day and night (GLMM results, *p* < 0.05; Supplementary Table 2). *Post-hoc* tests indicated that males woke up significantly more during the night than females (t = 3.190, *p* = 0.001), whereas females slept significantly more during the day (z = − 10.993, *p* < 0.001; Fig. 2). Older dogs also slept more during the day (t = 3.295, *p* = 0.001) and had more sleeping bouts than younger dogs (z = 4.493, *p* < 0.001). Sleep duration was not different between sexes at night, nor for the number of sleeping bouts during the day.

**Figure 3:**
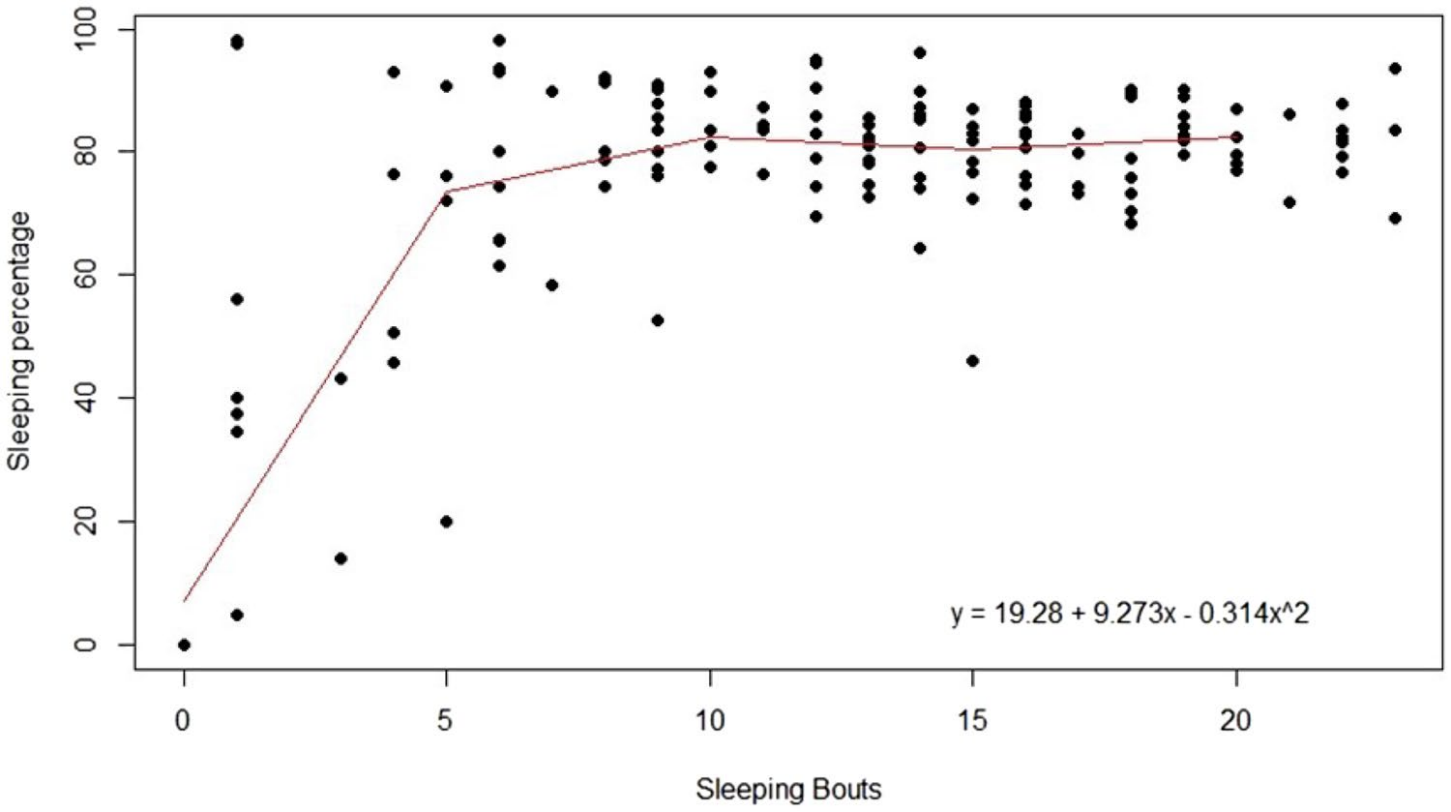
Sleeping percentage variation in response to the number of sleeping bouts in laboratory dogs. Trend line based on polynomial regression result: Percentage of sleep = 19.28 + 9.273(bouts) – 0.314(bouts)2, r2 = 0.66.

Sleep fragmentation (sleeping bouts) caused an increase in the percentage of time spent asleep during the night (Logistic regression, r^2^ = 0.66, F = 140, df = 144; Fig. 3.). Moreover, a GLMM analysis indicated that sleep duration in a previous night had a significant influence on the sleep duration on the following nights (F = 4.3409, df = 34, *p* < 0.001); the more fragmented the sleep in a previous night, the longer the duration of sleeping bouts in a subsequent night. However, the *post-hoc* test did not establish a temporal pattern, with only some specific nights affecting the subsequent nights. Thus, a temporal autocorrelation was performed to try to identify if the individuals’ sleeping patterns would affect the temporal distribution of sleep and the results showed that the total amount of time spent asleep did not influence sleep duration in following nights for the dogs (*p* > 0.05).

### Influence of sleep metrics on behavior

In general, the fragmentation of sleep increased inactivity during the day (t = 3.213, *p* < 0.001), increased time spent laying (z = 20.412, *p* < 0.001), and time spent standing inactive (z = 8.812, *p* < 0.001) during the following day. It also increased time spent eating (z = 2.488, *p* < 0.01), in locomotion (z = 11.037, *p* < 0.001), and increased the display of maintenance behaviors (z = 3.400, *p* < 0.001). Furthermore, fragmentation was associated with reduced expression of play behavior (z = − 2.042, *p* < 0.05), and reduction in the amount of time the dogs were alert during the day (z = − 5.333, *p* < 0.001). GLMM results are summarized in the Supplementary Table 2.

As expected, the amount of time dogs spent asleep during the day decreased the expression of most behaviors except for the time spent eating, which increased when dogs slept for longer (z = 2.020, *p* < 0.05). In contrast, the number of diurnals sleeping bouts had a mixed effect on behaviors. It increased the number of times dogs were recorded sleeping (z = 36.51, *p* < 0,001), or alert (z = 4,585, *p* < 0,001), decreased the amount of eating (z = − 5,797, *p* < 0,001) and it was the only variable, which influenced vocalizations, which decreased when the sleeping bouts were higher during the day (z = − 3.826, *p* < 0.001). GLMM results are summarized in Supplementary Table 3.

## Discussion

The mean time spent asleep at night for our dogs was significantly less (370 ± 232 min, 64.9%) than previously reported (e.g. 660 min for shelter dogs^26^; 720 min for laboratory dogs^31^). Moreover, the greatest difference observed was in the amount of time spent sleeping during the day. While other studies reported that dogs spent somewhere between 20 and 40% of daytime asleep^30,34^, our dogs slept only 6% of the time, and not all individuals exhibited this behavior. This pattern has also been observed for shelter dogs^26^. Considering that the most observed diurnal behavior in our study was inactivity, it is possible that the motivation for sleep is present, however, possibly due to external factors, the dogs could not sleep. Prior investigations concluded that dogs in kennels and shelters may suffer from compromised welfare due to noise and barking^50,51^. Furthermore, kennels practices have also been found to be a main source for sleep disturbances^26^. Hence, is likely that ongoing activities nearby and in the kennels are affecting our population. Even though barking accounted for only 5% of daily recordings for the studied individuals, it was mainly observed when nearby disturbances occurred (such as cars or people passing by or due to the presence of staff). Thus, further studies should assess the influence of the external environment on dogs in kennels or shelter conditions.

Our study also found that the number of sleeping bouts was different from what is normally seen in dogs. At first, the number of bouts seemed high (from 5 to 23), yet again, it was less than reported in the literature (e.g. average of 23, 32 and 60)^26,34,35^. Furthermore, it was possible to verify that the percentage of time spent asleep was directly related to the number of bouts, with the overall duration of sleep increasing when more bouts were present. This association can be explained by the occurrence of nights with less sleep fragmentation, it does not necessarily indicate longer hours of sleep, but instead that the dogs had fewer bouts (i.e., individuals spent more time awake before, after or in between bouts of sleep). Nonetheless, the greatest variation in sleep duration was mostly observed when dogs had less than 10 bouts per night with little variation in sleep percentage when 10 or more bouts occurred (Fig. 3).

Even so, the number of bouts is still less than expected for normal sleeping patterns in dogs and this may be connected to an altered sleep architecture due to the irregular patterns of sleep; since the dogs are not sleeping during the day, they slept more continuously and in longer bouts during the night, instead of having several bouts throughout night and day. In humans and other animals, sleep deprivation causes a rebound effect, with the motivation for sleep increasing when several episodes of deprivation have occurred^11^. Furthermore, prolonged wakefulness results in longer bouts of sleep and, consequently, decreases the number of spontaneous awakenings in subsequent episodes of sleep^9,27^. As the first episode of sleep at night regulates the body physiology to increase sleep motivation, we observed that most bouts (fragmentation) occurred early in the night, while in later hours dog slept for longer and only woke up to perform maintenance behaviors or to bark, similarly to other studies^36^.

Our findings suggest that female dogs had better rebounds of sleep and were able to maintain longer bouts, which in itself is a form of improved sleep quality^52^. The same has been observed in studies of humans and rats; after episodes of sleep deprivation females have the ability to recover more efficiently than males by displaying longer bouts of slow-wave sleep^40,41^. Thus, it is possible that male dogs were more affected by the effects of sleep deprivation, as biologically they were unable to restore sleep as efficiently as females. Although older dogs are known to sleep more when compared to younger dogs^31,34^, we did not find any significant differences between older and younger dogs’ sleep at night; though older dogs slept more during the day than younger ones. A result corroborated by another study investigating afternoon ‘naps’ in dogs^53^.

Inactivity was the behavior most observed in the diurnal observations of the dogs. Dogs are predominantly diurnal when housed in homes and kennels, meaning they will express more activity during the day and concentrate a higher proportion of sleep (especially REM-sleep) during the night^30,54^. Hence, it is not expected to observe elevated daytime inactivity as part of their normal behavior. As the dogs in our study displayed an altered sleep structure, we can identify a cyclic effect – the disruptive pattern of sleep at night makes them less motivated or able to remain active during the day, and, as consequence, the lack of activity during the day does not promote better sleep at night. This could also be the reason why our results displayed increased locomotion at the day as an effect of increased sleep fragmentation.

Previous literature elucidated a positive change in sleep structure after activities in dogs, however, the assessment was done over a short period of time^37^. In humans, regular physical activity, in opposed to extreme activity, is the most beneficial to promote sleep^55^. Thus, as inactivity remains the major behavior observed, the benefit of more movement (increased locomotion) does not appear to be enough to promote sleep. Similarly, play was also negatively correlated with sleep fragmentation. So far sleep has not been investigated in relation to play, but as this behavior is a form of exercise that arises due to excessive energy^56^, we could expect that sleep-deprived dogs would not spend energy playing.

Moreover, play has been associated with positive affective states and positive welfare, as it only arises when conditions are optimal^57^. Play also decreases stress responses in dogs. As individuals in our study experience a poor quality of sleep leading to a decrease in play, it also could be a sign of poorer welfare. Recently, a study demonstrated that sleep in pet dogs change on days with positive social interactions^39^. This could mean that behaviors that contribute to positive experiences, such as positive play, may contribute to better sleep. Despite fragmentation decreasing play response in our dogs, a positive association was found between play and sleep duration, which strengthens the assumption of a relationship between sleep and pay.

Alert behaviors were also negatively associated with increased sleep fragmentation. As aforementioned, the environment of the kennels has several stimuli that may be interfering with diurnal sleep in our population. In this case, perhaps the lack of attention is an attempt to mitigate some of these adverse conditions and try to rest, even if unsuccessfully.

Feeding was the only behavior that was equally associated with sleep at night and sleep during the day. Sleep latency, sleep loss and sleep dysfunctions have all been associated with changes in eating habits in humans and other animals, being considered catalysts to increased consumption and energy intake during the day and responsive for changes in the metabolism associated with increased body weight^58,59^. In dogs, the association between feeding behavior and sleep has been investigated before and results demonstrated that feeding times and frequency of feeding had a significant impact on sleep^60^. Shifting from a single feed to being fed twice daily resulted in an early onset of sleeping bouts at night, resulted in a shorter latency to sleep during the night and affected the number of bouts during the day, with the individuals sleeping for longer, but in fewer bouts. In our dogs, a peak of feeding was noticed in the morning, followed by small feeding events throughout the day, but with no pattern. Sleep loss reduces activity and increases sleepiness during the day, which also impacts the patterns of appetite^59,61^. Since most individuals also did not sleep during the day, it is possible that feeding has been disturbed by sleep suppression, but also, the absence of specific feeding times and amount of food consumed further contribute to changes in sleep. It is suggested that feeding patterns do influence locomotor behavior in dogs, and consequently, this will affect their sleep^30,31^.

Overall, the main findings of our study suggest that sleep fragmentation affects the expression of diurnal behaviors; however, this relationship should be interpreted with caution as the correlational nature of this research means that we cannot separate cause and effect in terms of how factors affect sleep. Presently, only one other study has attempted to unveil the impacts of sleep deprivation on dogs’ behavior and welfare and similarly has argued the limitation in determining cause and effect^26^. Nonetheless, results are comparable, as both studies indicated a reduction in sleeping time, increased fragmentation of sleep at night, and decreased activity during the day; in contrast to previous evidence arguing of a causal relationship between the outcomes of sleep deprivation and daytime behaviors^30,34^. Despite this limitation of a non-experimental study, we have found strong evidence that highlight the cyclic interactional effects of sleep quality and subsequent daytime behaviors on each other. The welfare implications of these effects will require further investigations; however, studies have shown that the kennel environment can be detrimental for dog welfare, especially long-term kenneling, which frequently leads to chronic stress, depletion of the immune system and increased inactivity, associated with apathy ^62,63^. Thus, sleep quality should be considered in management practices, as the cyclic effect observed in our study could be caused by and be contributing to the increase in negative responses associated with the kennel environment.

## Conclusion

Dogs in our study slept substantially less than previously reported in the literature. Moreover, the sleep architecture is different from the patterns observed in other studies, with fewer awakenings during the night and almost no sleep recorded during the day. Additionally, sleep was different between sexes, but not in age groups during the night. We also observed that quantity and quality of sleep directly affected the dogs’ daytime behavior, which became more inactive, ate more, played less, and were less alert. Sleep fragmentation appears to leave the animals tired and less responsive to their environment, remaining mostly inactive during the day. Even with locomotion increasing following highly fragmented sleep, the amount of activity was not sufficient to, subsequently, increase sleep duration. Our findings suggest that sleep deprivation can affect daytime behaviors in laboratory dogs and further studies may help to unveil the relationship between the effects of sleep loss and behavioral patterns. In addition, husbandry practices and the kennels’ environment appear to be contributing to the altered sleep behavior. Changes in behavioral patterns are associated with individuals' welfare and can be used to measure adaptability to the environment. Consequently, the altered behavior related to sleep loss may indicate compromised welfare for the dogs in our study. Thus, appropriate measures should be taken to mitigate shortcomings, such as the improvement of housing conditions, the use of enrichment practices and a regular exercise routine, to ensure dogs’ sleep quality and overall welfare are met. Overall, monitoring sleep has the potential to be used as a measure of animal welfare.

## Methods

### Ethical statement

This study was approved by the Science & Technology Research Ethics Panel of the University of Salford Manchester (STR1617-80) and by the Animal Ethics Committee of the Federal University of Ouro Preto, Minas Gerais, Brazil (Protocol 2017/04). Dogs were maintained following the guidelines of the National Animal experimentation Control Council, Normative Resolution nº12^64^, however, the standard of care is not comparable to current legislation in other countries. Authors in no way endorse or condone the reported animal practices and the investigation aimed to understand the welfare of dogs using sleep disruption as a proxy measure of animal welfare. No changes to their routine or environment were made for this study.

### Subjects and study site

Seven males and six female mix-breed adult dogs (5.9 ± 1.8 years old, mean ± SD) were randomly selected from a population of 20 dogs from the laboratory kennels at the Centre for Animal Science, in the University of Ouro Preto, State of Minas Gerais, Brazil.

Dogs were paired housed in outdoor kennels, separated into sections by sex. All kennels had a basic rectangular layout (5.8 m × 1.6 m × 1.65 m) with bare concrete walls and flooring, and one-third of the space was covered for shelter. The females´ kennels also had a small room at the back, which was used as a birthing den when needed. Dogs could freely see others through the kennels front gate, but direct contact outside the pair was only possible during play/exercise sessions and only between animals of the same sex to avoid unnecessary breeding. Kennels had natural lighting (12–12 h light period; no artificial lighting was provided) and ambient temperature (mean: 18.3 ± 2.1 °C). Dogs had access to water and food ad libitum, which were replenished if necessary, during cleaning routines. Dogs were inspected regularly and were considered clinically healthy and behaving clinically normally throughout the study. No dogs were participating in any other research nor were bred while data collection took place.

### Data collection of behavioral data

Eight CCTV cameras with night vision capability (Swann SWDVK-845504) were installed in the kennels (two by kennel, four kennels in total) and positioned to ensure full coverage of the area. In the females´ kennels, one camera was placed inside a separate room. Dogs were monitored from October 2017 to May 2018, in a continuous 24-h, five-day assessment period. A total of 1560 h of video were collected, comprising 20 days and nights for each kennel spread equally in 5-day/24 h assessments for each dog (due to logistics not all dogs were assessed in the same week).

Behavioral data were coded as two separate periods: diurnal (07:00–17:59 h) and nocturnal (18:00–06:59 h) based on natural light levels. Diurnal behavior was recorded using focal sampling with instantaneous recordings of behavior at a 30-s interval^65^. Dogs were assessed randomly each hour, for 15 min, totalling 30 recordings per hour. The behaviors were classified using an ethogram for dogs (Supplementary Table S1), based on the literature^66,67^. Furthermore, if the dogs slept, the duration and the number of bouts were recorded. The data was coded using the software Boris v.7.0.12 (Behavioral Observation Research Interactive Software^68^).

Nighttime behaviors were recorded using focal sampling with continuous recordings of behavior^65^. Due to the quantity of data that continuous observation generates, nocturnal behaviors were allocated into three broad categories: sleep, rest, and activity. Additionally, we scored the number of sleeping bouts per night, the latency until the first sleeping bout and the last sleeping bout before the diurnal observations period began.

### Statistical analysis

Behavioral data were tested for normality using the Anderson–Darling test and found to be non-parametric. Descriptive statistics were calculated for all metrics and results are reported as either counts or percentages with standard deviation. Differences in the amount of time spent asleep and the number of sleeping bouts during day and night were investigated using a Wilcoxon signed ranked test. Differences among the categories of activity were verified using a Friedman test with Dunn’s *Post-hoc*.

A regression analysis was used to verify the association between sleep duration and sleep fragmentation. Residuals were evaluated with a Scatterplot and found to be non-parametric, therefore a polynomial regression was chosen as the appropriate model. After transforming the data, the best-adjusted curve was found to be the one in a polynomial regression with quadratic transformation (variation of Y is explained by the variation of X^2^).

The relationships between sleeping metrics such as sleeping bouts or sleeping duration (response variables) and other variables, such as age and sex (explanatory variables) were analyzed using generalized linear mixed effect models (GLMMs, *lmer* function for data with normal distribution and *glmer* for non-normal data, both with *lme4* package in R^69,70^). To further investigate if the fragmentation of sleep affected subsequent nights of sleep, a Time Series Analysis using a Temporal Autocorrelation was conducted. This analysis correlates observations of a time series separated by n units of time^69^.

Further GLMM models were constructed to understand how sleep fragmentation and sleep duration (explanatory variables) affected the dogs’ behavior (response variables). Due to the scale difference of each of the variables included in the models (e.g. sleeping duration in seconds vs total count of behavior), the data were scaled to reduce size difference and avoid overdispersion of residuals^71^. Variables’ significance was determined by model comparison and backward selection until a minimum suitable model was reached. Due to our daily repeated assessments (i.e., longitudinal data, *sensu*^69^), we fitted the sampling day as a random effect varying in the intercept (1|day)^70^. Significant differences between expressed behaviors were then identified using pair-wise comparisons^69^. All GLMMs models were submitted to residual analysis. All statistical analyses were carried out using R Studio. All data generated or analyzed during this study are available at the Mendeley Data website (https://doi.org/10.17632/7nnwc3f3kv.1).

### Additional information

Raw data used in the analysis is available online (Mendeley Data, V1, https://doi.org/10.17632/vfd7m2x38k.1).

## Supplementary Information

Supplementary Information.
