# Supplementary material for: The cyclic interaction between daytime behavior and the sleep behavior of laboratory dogs
Source: Sci Rep. 2022 Jan 10;12:478. doi: 10.1038/s41598-021-04502-2 (PMC8748904; doi:10.1038/s41598-021-04502-2)
Supplement: Supplementary file 1 — Supplementary Information. [file 41598_2021_4502_MOESM1_ESM.docx]

Table S1. Definition of behavioral categories used to assess diurnal and nocturnal behaviors of dogs.


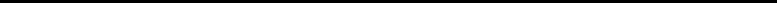


Behavioral states* Definition

| **Consumption** | Feed | Dog is actively consuming food. |
| --- | --- | --- |
|  | Drink | Dog is actively consuming water. |
| **Interaction** | Explore | Dog interacts with the environment approaching stimuli in an  investigative manner (smell, scratch, lick, touch, or dig). |
| **Locomotion** | Locomotion | Dog is moving from one location to another with alternate limbs |
|  |  | touching the ground at different times throughout at any |
|  |  | velocity. |
| **Rest** |  | Dog is laying down, stationary, with abdomen touching the |
|  | Resting | ground with either dorsal, lateral, or caudal side. Limbs can be  stretched in the front, laterally or curled near the body. Eyes |
|  |  | open and movement of ears can be observed. |
|  |  | Dog has similar positions as resting, but eyes remain closed for |
|  | Sleep | at least 02 minutes. Paddling of limbs, rapid eye movement and |
|  |  | occasional vocalizations can be expressed. |
| **Stationary** |  | Dog is in an upright position with all limbs extended. Ears are in |
|  | Alert | upright position. Ears and head are pointing into the direction |
|  |  | of stimuli. |
|  | Sitting | Dog is in an upright position, with hind legs flexed while front |
|  | inactive | legs are extended straight. |
|  | Stand | Dog is in upright position with all limbs extended supporting the |
|  | inactive | body while immobile. |
| **Social** | Social | Dog is interacting (rub, smell other, present or groom other) |
|  | Affiliative | with a conspecific in an affiliative manner. |
|  | Social | Dog is interacting (bite, chase, strike with paw, show teeth, hair |
|  | Aggressive | bristle) with a conspecific in an aggressive manner. |
|  |  | Dog engages with a conspecific, less than two body lengths |
|  | Play | away, and different behaviors are observed (jump over other, |
|  |  | chase, pulling tail, mouth, roll). |

| **Maintenance** | Groom | Dog grooms itself using its tongue, teeth, or mouth. |
| --- | --- | --- |
|  | Excrete | Dog releases faeces or urine from the body. |
| **Repetitive**** | Tail- chasing | A quick and repetitive circular motion, where the dog attempts to bite its tail. |
|  | Pacing | A locomotor movement with the animal traversing the same  pathway at least twice in quick succession. |
| **Panting**** |  | Dog’s mouth is open, tongue out. Dog quickly inhales and  exhales through mouth breathing. Chest breathing movements are visible. |
| **Vocalization**** |  | Dog opens and closes mouth while emitting repetitively short and constant vocalizations that can vary in frequency from high  to deep and low. |
|  | Rhythmic  Barking |  |

* Based on^62^; **Based on^63^.

**Table S2. Linear Mixed Effects Models (GLMM) results for the optimal models describing the relationship laboratory dogs´ age, sex and sleep metrics (independent variables).**

| **Response** | **Parameters** | **Estimate ± SE** | **Coefficient value** | **p** | **Error distribution** |
| --- | --- | --- | --- | --- | --- |
| Sleep bouts (night) | Intercept | 11.3513 ± 0.831 | 1.144 | ns | Gaussian |
|  | Age | 0.2815 ± 0.3118 | 0.903 | ns |  |
|  | SexMale^a^ | 4.5118 ± 1.4163 | 3.190 | 0.001** |  |
| Sleep bouts (Day) | Intercept | -2.9472± 0.3271 | -9.009 | 2e^-16^*** | Poisson |
|  | Age | 0.2033± 0.0453 | 4.493 | 7.03e^-16^*** |  |
|  | GenderMale^a^ | -0.2331± 0.1917 | -1.216 | ns |  |
| Sleep duration (day) | Intercept | -0.1955± 0.1307 | -1.495 | ns | Gaussian |
|  | Age | 0.0637± 0.0913 | 3.295 | 0.001** |  |
|  | GenderMale | -0.0507± 0.0869 | -0.583 | ns |  |
| Sleep Count (day) | Intercept | -1.4220± 0.4323 | -3.289 | 0.001** | Poisson |
|  | Age | 0.2334± 0.0167 | 13.979 | 2e^-16^*** |  |
|  | GenderMale^a^ | -0.7909± 0.0719 | -10.993 | 2e^-16^*** |  |

**Table S3. Linear Mixed Effects Models (GLMM) results for the optimal models describing the relationship between laboratory dogs’ behaviours and sleep metrics (independent variables).**

| **Behaviour** |  | **Independent variable** | **Estimate ± SE** | **Coefficient**  **value** | **p** | **Error**  **distribution** |
| --- | --- | --- | --- | --- | --- | --- |
| Alert |  | Sleep bouts (night) | -0.0529 ± 0.0099 | -5.333 | 9.68e^-08^ | Poisson |
|  |  | Sleep bouts (day) | 2.6647 ± 0.5811 | 4.585 | 4.54e^-06^ |  |
|  |  | Sleep duration (day) | -8.2486 ± 1.9818 | -4.162 | 3.15e^-05^ |  |
| Eating |  | Sleep bouts (night) | 0.0243 ± 0.0097 | 2.488 | 0.012 | Poisson |
|  |  | Sleep bouts (day) | -1.5813 ± 0.2728 | -5.797 | 6.75e^-09^ |  |
|  |  | Sleep duration (day) | 0.1431 ± 0.0099 | 2.020 | 0.043 |  |
| Inactive |  | Sleep bouts (night) | 0.0597 ± 0.0185 | 3.213 | 1.31e^-03^ | Gaussian |
| Lay Down |  | Sleep bouts (night) | 0.0383 ± 0.0025 | 15.294 | < 2e^-16^ | Poisson |
|  |  | Sleep bouts (day) | 0.4927 ± 0.0262 | 18.786 | < 2e^-16^ |  |
|  |  | Sleep duration (day) | -0.0002 ± 0.0000 | -6.657 | 2.8e^-11^ |  |
| Locomotion |  | Sleep bouts (night) | 0.0426 ± 0.0039 | 11.037 | < 2e^-16^ | Poisson |
|  |  | Sleep bouts (day) | -0.7863 ± 0.1042 | -7.550 | 4.36e^-14^ |  |
|  |  | Sleep duration (day) | -0.0974 ± 0.0453 | -2.151 | 0.031 |  |
| Maintenance |  | Sleep bouts (night) | 0.0266 ± 0.0078 | 3.400 | 6.75e^-4^ | Poisson |
|  |  | Sleep bouts (day) | 0.1326 ± 0.0900 | 1.473 | ns |  |
|  |  | Sleep duration (day) | 0.0327 ± 0.0327 | 0.858 | ns |  |
| Play |  | Sleep bouts (night) | -0.0397 ± 0.0194 | -2.042 | 0.041 | Poisson |
|  |  | Sleep duration (day) | -3.6741 ± 2.4396 | -1.506 | 0.132 |  |
| Sleeping (day) |  | Sleep bouts (night) | -0.0115 ± 0.0068 | -1.706 | ns | Poisson |
|  |  | Sleep bouts (day) | 1.1478 ± 0.0314 | 36.521 | < 2e^-16^ |  |
|  |  | Sleep duration (day) | 0.2570 ± 0.0126 | 20.348 | < 2e^-16^ |  |
| Sitting |  | Sleep bouts (night) | 0.0028 ± 0.0104 | 0.269 | ns | Poisson |
|  |  | Sleep bouts (day) | -0.3648 ± 0.1888 | -1.933 | 0.053 |  |
|  |  | Sleep duration (day) | -0.2914 ± 0.0990 | -2.943 | 0.003 |  |
| Standing |  | Sleep bouts (night) | 0.0303 ± 0.0034 | 8.821 | < 2e^-16^ | Poisson |
|  |  | Sleep bouts (day) | -0.5434 ± 0.0952 | -5.707 | 1.15e^-08^ |  |
|  |  | Sleep duration (day) | -0.2084 ± 0.0614 | -3.393 | 0.001 |  |
| Vocalizing |  | Sleep bouts (night) | 0.0039 ± 0.0137 | 0.287 | ns | Poisson |
|  |  | Sleep bouts (day) | -0.7979 ± 0.3449 | -2.313 | 0.027 |  |
|  |  | Sleep duration (day)  *p ≤ 0.05, **p ≤ 0.01, ***p ≤ 0.001. | 0.0847 ± 0.1452 | 0.584 | ns |  |


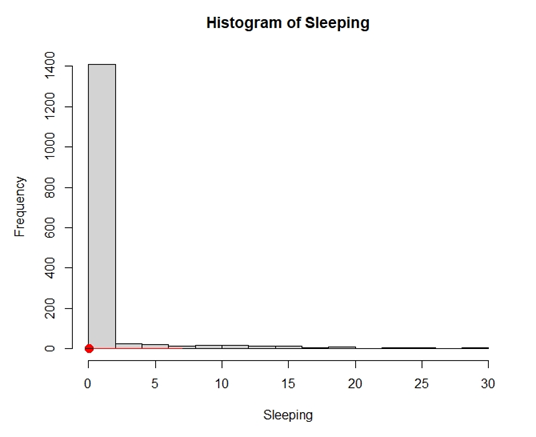


**Figure S1. Dispersion of observed data points (N=1217) recorded as day sleeping (counts) observed for female and male dogs in randomized 15-minutes observations with 30-seconds recording intervals. 0 = no sleep.**
